# Endoscopic transcanal management of incus long process defects: rebridging with bone cement versus incus interposition

**DOI:** 10.1007/s00405-022-07489-2

**Published:** 2022-06-18

**Authors:** Waleed Moneir, Mohammed Abdelbadie Salem, Ahmed Hemdan

**Affiliations:** Department of Otolaryngology, Mansoura Faculty of Medicine, Mansoura University, El-gomhoria street, Mansoura, Egypt

**Keywords:** Endoscopic transcanal ossiculoplasty, Glass ionomer bone cement, Incus interposition, Chronic suppurative otitis media

## Abstract

**Objectives:**

to compare hearing outcomes between endoscopic transcanal rebridging with bone cement and endoscopic transcanal incus interposition in patients with incus long process defects secondary to chronic suppurative otitis media (inactive mucosal type).

**Methods:**

This retrospective study was performed on 83 ears of 83 consecutive patients with incus long process defects secondary to chronic suppurative otitis media (inactive mucosal type). According to the extent of incus long process erosion and subsequent ossiculoplasty technique, patients were divided into 2 groups. Patients in group 1 had erosion involving up to two thirds of the length of the incus long process and underwent endoscopic transcanal rebridging with bone cement. Patients in group 2 had erosion involving more than two thirds of the length of the incus long process and underwent endoscopic transcanal incus interposition.

**Results:**

Hearing gain (mean ± standard deviation) was 21.39 ± 2.15 dB in group 1 and 19.71 ± 6.12 dB in group 2. A significantly greater hearing gain was achieved in bone cement group than in incus interposition group (*P* value < 0.001). Successful hearing outcome (post-operative air bone gap closure within 20 dB) was achieved in 81.6% and 71.1% of patients of group 1 and group 2 respectively.

**Conclusion:**

Endoscopic transcanal rebridging with bone cement offers greater hearing gain than endoscopic transcanal incus interposition. The two techniques remain reliable and cost-effective techniques in management of patients with incus long process defects. The main limitation of this study was the short follow-up period. Further studies with relatively long-term follow-up are strongly recommended.

## Introduction

Along the ossicular chain, the long process of the incus is the most vulnerable part for necrosis secondary to both trauma and infections [1, 2]. This will lead to incudostapedial joint discontinuity that results in conductive hearing loss [3]. Various techniques had been described to reconstruct such discontinuity including interposition of either autograft or homograft, partial ossicular replacement prosthesis (PORP) and rebridging with bone cement [4].

Throughout the literatures, different studies were carried out to compare incus interposition with rebridging with bone cement [5–11]. They all had a common feature where all surgeries were performed using the microscope. Since endoscopic transcanal ossiculoplasty is an evolving technique that has gained popularity in the last few years, we aimed to compare between endoscopic transcanal rebridging with bone cement and endoscopic transcanal incus interposition in management of incus long process defects. To our knowledge, this is the first study in the literatures that compared these two ossiculoplasty techniques endoscopically.

## Patients and methods

This study was a retrospective study carried out on 83 ears of 83 consecutive patients with eroded incus long process secondary to chronic suppurative otitis media (inactive mucosal type). All patients underwent endoscopic transcanal ossiculoplasty between March 2016 and March 2021 in our tertiary referral center. Institutional Ethics Committee approval was obtained prior to conduction of the study (code number: R.21.10.1481). Informed written consents were obtained prior to surgeries.

All involved patients had eroded incus long process secondary to chronic suppurative otitis media (inactive mucosal type). Selection of the type of ossiculoplasty; whether rebridging with bone cement or incus interposition; depended on the extent of erosion of the incus long process because rebridging with bone cement is not recommended for large defects involving more than two thirds of the length of the incus long process [7]. Accordingly, patients were divided into 2 groups. Patients in the first group (Group 1) had erosion involving up to two thirds of the length of the incus long process and underwent endoscopic transcanal incudostapedial rebridging with bone cement. Patients in the second group (Group 2), on the other hand, had erosion involving more than two thirds of the length of the incus long process and underwent endoscopic transcanal incus interposition. The cause of erosion of the incus long process was chronic suppurative otitis media (non cholesteatomatous; inactive mucosal type). Patients with cholesteatoma, post-traumatic disrupted incudostapedial joint, eroded stapes and/or malleus, failed tympanic membrane grafting were all excluded. Patients who lost to follow up were also excluded.

Audiologic evaluation was performed 6 months postoperatively in accordance with the American Academy of Otolaryngology- Head and Neck Surgery Committee on Hearing and Equilibrium guidelines for evaluation of the results of treatment of conductive hearing loss. Preoperative and postoperative air and bone conduction thresholds (AC and BC) were recorded at frequencies of 0.5, 1, 2, and 3 kHz. The air and bone conduction thresholds for 2 kHz and 4 kHz were averaged to estimate the 3 kHz results. The air bone gap (ABG) was calculated by measuring the difference between AC and BC thresholds [12].

### Surgical technique

A 0° rigid endoscope, 4 mm in diameter and 17 cm in length (Karl Storz, Germany) connected to a high-definition camera head and monitor (Karl Storz H3-Z TH100, Tuttlingen, Germany) together with the standard endoscopic ear surgery (EES) instruments were utilized. All surgeries were performed under general anesthesia. The external ear canal was infiltrated with 1/100,000 epinephrine. Using a Rosen needle (Karl Storz, Germany), circumferential trimming of the margin of the perforation was performed to allow complete de-epithelization of the remnant of the tympanic membrane. An incision in the posterior canal wall was made 6–8 mm lateral to the annulus. Elevation of the tympanomeatal flap was then performed till the fibrous annulus, which was then raised out of its bony groove (sulcus). Epinephrine-soaked cottonoids (1:100,000) were used to control bleeding during tympanomeatal flap elevation. The chorda tympani nerve was then mobilized, and the middle ear was entered. Posterosuperior bony canal wall was curetted to enhance exposure of the incudostapedial complex. Ossicular chain reconstruction; either by rebridging with bone cement or incus interposition; was performed. The continuity of the new ossicular system was tested by gentle palpation of the malleus (with a needle), while observing the movements of the stapes and round window membrane. Temporalis fascia graft was harvested and used for grafting the tympanic membrane. Gelfoam^®^ was placed medial and lateral to the graft (after repositioning the tympano-meatal flap). Ear wick was inserted in the external ear canal and finally dressing was applied.

### Group 1 (*n* = 38)

**Fig. 1:**
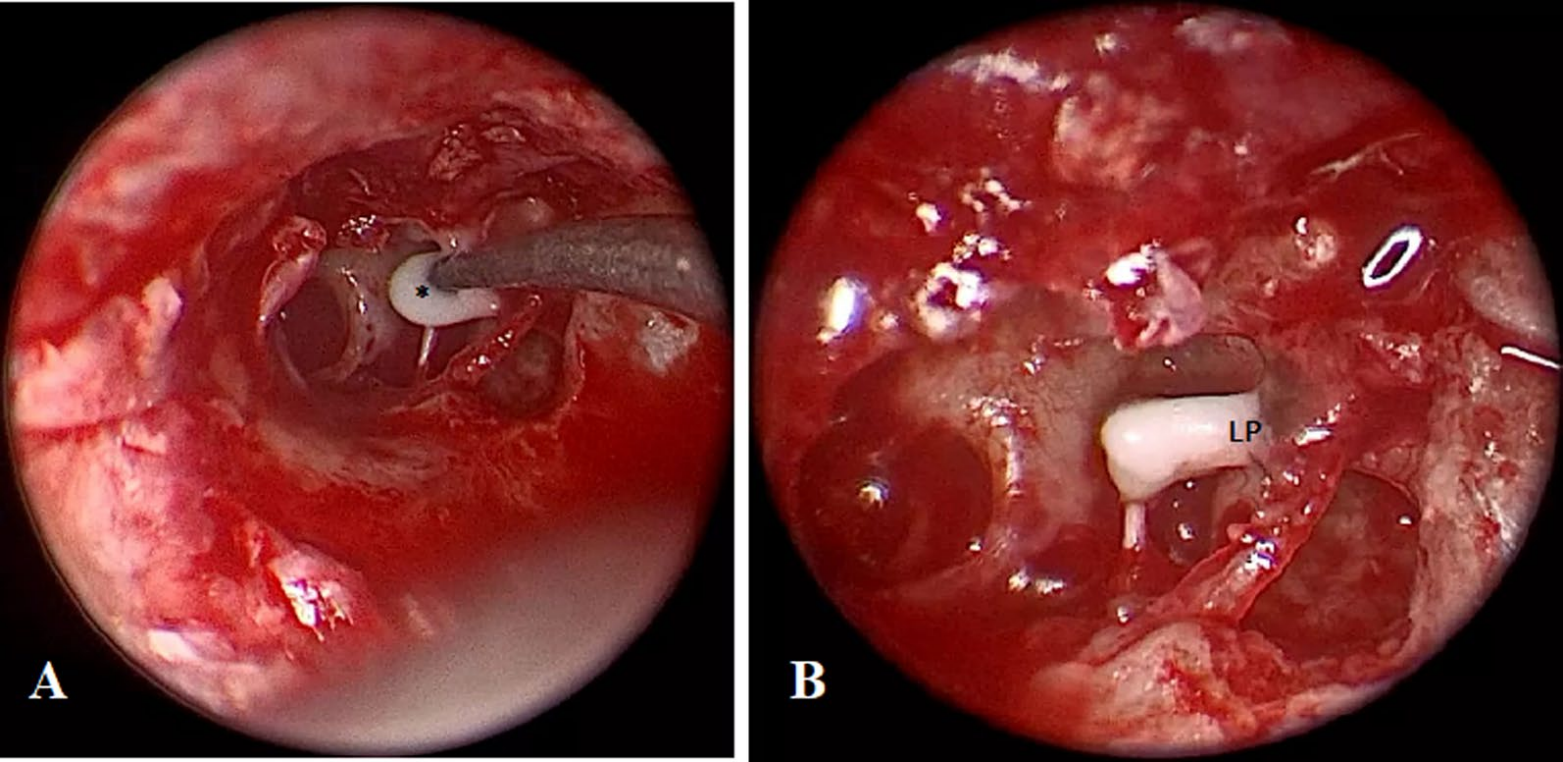
**A** Glass ionomer bone cement was applied drop by drop (with a needle) into the space between the stapes head and incus (*: drop of bone cement). **B** Bone cement was left undisturbed till it hardened. *LP* incus long process

Meron™ (Voco, Germany, 1,118,221), a glass ionomer luting cement, was used in this group for incudostapedial rebridging. Before bone cement was applied, we removed all mucosal remnants and blood overlying the incus long process and the stapes head to obtain the dry bony surfaces required for good adherence of the bone cement. As described in manufacturer instructions; one level measuring scoop of powder (composed of glass powder, polycarboxylic acid), and one drop of prepackaged liquid were put on a glass plate and mixed for 30 s till the mixture became homogeneous. To prevent unintentional contamination of the middle-ear, small pieces of Gelfoam^®^ were led down into the middle-ear cavity. When the mixture became muddy (a paste-like consistency), it was applied drop by drop into the space between the stapes head and incus (Fig. 1A). After the bone cement was applied, it developed a bony-like consistency after 5–10 min and lost its shiny appearance (Fig. 1B). After hardening, the movement of the ossicles was checked.

### Group 2 (n = 45)

**Fig. 2:**
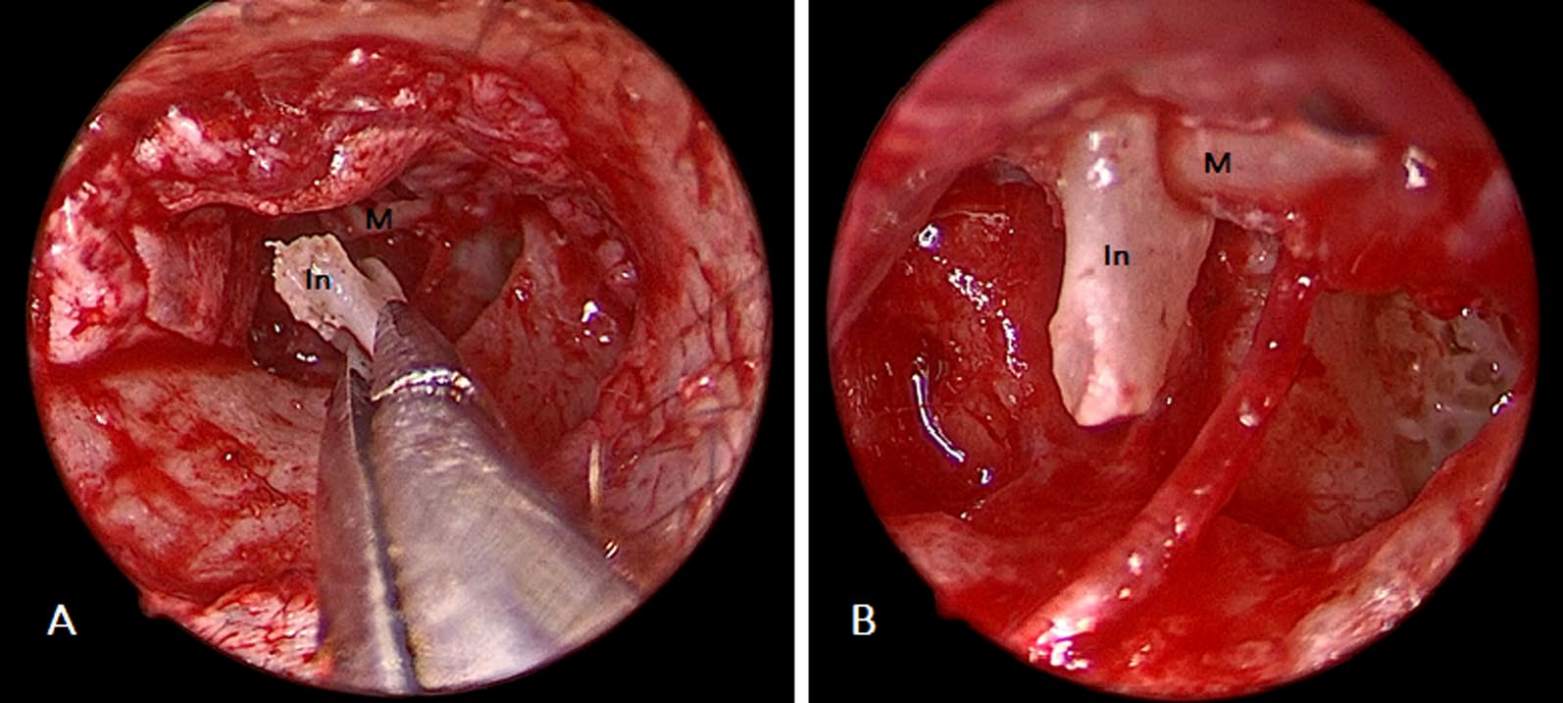
**A** Neo-incus was reintroduced into the middle ear. **B** Neo-incus was fitted between the stapes head and the malleus handle. *In* neo-incus, *M* malleus

The incus body was disarticulated from the malleus head and extracted. Using a 1.0- and 0.8-mm diamond drill, sculpturing of the incus was performed. The neo-incus was then reintroduced into the middle ear (Fig. 2A) and using a small right-angled hook, the neo-incus was fitted between the stapes head and the malleus handle (Fig. 2B). Care was taken to place the neo-incus properly between the malleus and stapes, without excessive tension or wedging. Finally, Gelfoam^®^ was used to stabilize the neo-incus in position till development of fibrous union and to support tympanic membrane fascial graft.

### Follow -up

The first follow up visit was scheduled 1 week postoperatively for removal of the covering aural gauze, sutures and the ear wick. In addition, otoscopic examination and tuning fork hearing tests were performed. Antibiotic ear drops were started at the first follow up visit and continued for 1 week. The second visit was scheduled 1 month postoperatively to assess graft healing and to exclude the presence of any possible complications. Audiologic evaluation was performed after 6 months (pure tone audiometry and speech audiometry).

### Data collection and statistical analysis

Preoperative assessment and postoperative follow up were recorded, tabulated, analyzed and discussed. Analysis of the results was performed using SPSS for Windows version 28. statistical software program (Statistical Package for Social Sciences = SPSS Inc., Chicago, IL, USA). The paired *t*-test was utilized to compare preoperative with postoperative results. To compare the results of the two groups, the chi-square test was used. Statistically significant results were considered at *P* values < 0.05. Results were presented as mean ± standard deviation (SD).

## Results

Ninety-three patients were enrolled in the study. Rebridging with bone cement was performed in 43 patients (group 1) while incus interposition was performed in the other 50 patients (group 2) (Table 1). Seven patients (3 in group 1 and 4 in group 2) had postoperative failed tympanic membrane grafting and were excluded (Table 1). Three patients (2 in group 1 and 1 in group 2) lost to follow up and were also excluded. After exclusion of all these patients, 38 patients remained in group 1 and 45 in group 2 (Table 1). Group 1 comprised 16 male and 22 female patients with a mean age of 32.21 ± 12.26 years (range 13–56 years) (Table 1). Group 2, on the other hand, included 20 male and 25 female patients with a mean age of 31.78 ± 12.54 years (range 12–58 years) (Table 1).

**Table 1:** Number of patients, gender and age

	Group 1	Group 2
Total number of patients underwent surgery	43	50
Excluded patients		
Failed tympanic membrane grafting	3	4
Lost to follow up	2	1
Final number of patients involved in the study	38	45
Gender		
Females	22	25
Males	16	20
Age		
Mean ± SD	32.21 ± 12.26 years	31.78 ± 12.54 years
Range	13–56 years	12–58 years
No of patients with postoperative taste disturbance	2	3

No serious complications; in the short term; were encountered apart from taste disturbance which was observed in 2 and 3 patients in group 1 and group 2 respectively (Table 1).

Patients were followed up for at least 6 months. The mean follow-up period in group 1 was 17.67 ± 5.40 months while in group 2, it was 16.76 ± 6.64 months.

The mean preoperative AC thresholds for group 1 and group 2 were 60.05 ± 11.60 dB and 62.01 ± 12.04 dB respectively with statistically non-significant difference (*P* value 0.16). The mean postoperative AC thresholds for group 1 and group 2 were 38.66 ± 10.13 dB and 42.89 ± 8.40 dB respectively with statistically significant difference (*P* value < 0.001). In each group, there was a statistically significant difference between preoperative and postoperative AC thresholds (*P* value < 0.001 in each group) (Table 2). The mean preoperative BC thresholds for group 1 and group 2 were 22.03 ± 5.59 dB and 22.88 ± 5.79 dB respectively with statistically non-significant difference (*P* value 0.47). The mean postoperative BC thresholds for group 1 and group 2 were 21.95 ± 5.37 dB and 22.82 ± 5.90 dB respectively with statistically non-significant difference (*P* value 0.16). In each group, there was statistically non-significant difference between preoperative and postoperative BC thresholds (*P* values 0.26 and 0.16 respectively) (Table 2). The mean preoperative ABG thresholds for group 1 and group 2 were 38.03 ± 6.06 dB and 39.14 ± 6.28 dB respectively with statistically non-significant difference (*P* value 0.19). The mean postoperative ABG thresholds for group 1 and group 2 were 16.71 ± 4.52 dB and 20.07 ± 4.31 dB respectively with statistically significant difference (*P* value < 0.001). In each group, there was a statistically significant difference between preoperative and postoperative ABG thresholds (*P* value < 0.001 in each group) (Table 2). The mean preoperative speech reception thresholds (SRTs) for group 1 and group 2 were 60.39 ± 11.17 dB and 62.22 ± 11.65 dB respectively with a statistically non-significant difference (*P* value 0.32). The mean postoperative SRTs were 39.74 ± 11.02 dB and 44 ± 8.37 dB in group 1 and group 2 respectively with a statistically significant difference (*P* value < 0.001). In each group, there was a statistically significant difference between preoperative and postoperative SRTs (*P* values < 0.001 in each group) (Table 2). The mean preoperative speech discrimination scores (SDS) for group 1 and group 2 were 97.82 ± 5.44 and 97.56 ± 5.37 respectively with a statistically non-significant difference (*P* value 0.18). The mean postoperative SDSs were 97.96 ± 5.16 and 97.64 ± 5.10 in group 1 and group 2 respectively with a statistically non-significant difference (*P* value 0.24). There was a statistically non-significant difference between preoperative and postoperative SDS in each group (*P* values 0.32 and 0.16 in group 1 and group 2 respectively) (Table 2). The mean hearing gain in group 1 was 21.39 ± 2.15 dB dB and in group 2 was 19.71 ± 6.12 dB. Hearing gain was significantly greater in bone cement group than in incus interposition group (*P* value < 0.001). Successful hearing outcome (ABG closure within 20 dB) was achieved in 81.6% and 71.1% of patients of group 1 and group 2 respectively.

**Table 2:** Comparison between groups 1 and 2 as regard mean AC, BC, ABG and hearing gain

	Group 1	Group 2	*P* value
AC threshold (mean ± SD)			
Preoperative	60.05 ± 11.60	62.01 ± 12.04	0.16
Postoperative	38.66 ± 10.13	42.89 ± 8.40	< 0.001
*P* value	< 0.001	< 0.001	
BC threshold (mean ± SD)			
Preoperative	22.03 ± 5.59	22.88 ± 5.79	0.47
Postoperative	21.95 ± 5.37	22.82 ± 5.90	0.16
*P* value	0.26	0.16	
ABG (mean ± SD)			
Preoperative	38.03 ± 6.06	39.14 ± 6.28	0.19
Postoperative	16.71 ± 4.52	20.07 ± 4.31	< 0.001
*P* value	< 0.001	< 0.001	
SRT (mean ± SD)			
Preoperative	60.39 ± 11.17	62.22 ± 11.65	0.32
Postoperative	39.74 ± 11.02	44 ± 8.37	< 0.001
*P* value	< 0.001	< 0.001	
SDS (mean ± SD)			
Preoperative	97.82 ± 5.44	97.56 ± 5.37	0.18
Postoperative	97.96 ± 5.16	97.64 ± 5.10	0.24
*P* value	0.32	0.16	
Hearing gain (mean ± SD)	21.39 ± 2.15	19.71 ± 6.12	< 0.001

## Discussion

When erosion of the incus long process is encountered; in the presence of intact stapes and malleus; two options become available: rebridging the ossicular gap or bypassing the defect [13].

Various materials have been utilized for bypassing such defects. They are generally divided into autografts, homografts and allografts [15]. The use of homografts is abandoned in many countries due to the risk of transmission of infectious diseases [6]. Ossicles, cortical bone, and cartilage may be utilized as autografts. They have the advantages of biocompatibility, low extrusion rate, cost-effectiveness (as they are readily available in surgical field) and absence of risk of transmission of infection. Their disadvantages, on the other hand, include necrosis, adhesion and displacement [6, 15]. Allografts are made from synesthetic biocompatible materials (e.g., Teflon, ceramics, porus plastics, titanium, gold…etc.). Such allografts, including partial ossicular replacement prosthesis (PORP), have the advantages of being ready for use without the need for drilling or shaping. However, their disadvantages include high excursion rate, relatively high cost, prosthesis displacement and ossicular necrosis secondary to contact with the prosthesis [5, 15].

Bypassing the incudostapedial joint defects by incus interposition is performed by reshaping the extracted incus followed by its insertion between the handle of malleus and the stapes head [14]. Such procedure was classically performed using the microscope. Throughout the literatures, different studies had evaluated hearing outcome after microscopic incus interposition. O’Reilly et al. performed microscopic incus interposition in 137 patients and attained a mean postoperative ABG of 18.6 dB with 66.4% of the patients showing ABG closure within 20 dB [15]. Albu et al. recorded a mean ABG of 14.58 dB after microscopic incus interposition [16]. Faramarzi et al. performed microscopic incus interposition in 199 ears and obtained a mean postoperative ABG of 16 dB with ABG closure within 20 dB in 78.9% of patients [17].

Rebridging ossiculoplasty with glass ionomer bone cement has various advantages including preservation of the normal anatomy and physiology of the ossicular chain that results in satisfactory hearing outcome. In addition, it is easily prepared and applied [18]. The main limitation of glass ionomer bone cement is its neurotoxicity which can be avoided by introducing small pieces of gel foam before its application [19]. If inadvertent contamination occurs, bone cement should be aspirated followed by irrigation of the middle-ear cavity with serum [20]. As in incus interposition, most studies had utilized the microscope in rebridging with bone cement. Babu and Seidman reported a mean postoperative ABG of 10 dB in 18 patients who underwent microscopic rebridging with bone cement with ABG closure within 20 dB in 17 out of the 18 patients [21]. Brask performed microscopic rebridging with bone cement in 22 patients and achieved a mean post-operative ABG of 16 dB, with an ABG closure within 20 dB in 81.3% of the patients [22]. Ozer et al. performed microscopic rebridging with bone cement in 15 patients and reported a mean postoperative ABG of 14.3 dB with ABG closure within 20 dB in 9 out of the 15 patients [23].

Despite the increasing interest in transcanal endoscopic ear surgery (TEES), few studies about endoscopic transcanal ossiculoplasty had been published [24]. Iannella performed endoscopic interposition in 6 patients and achieved a mean postoperative ABG of 14.7 dB [25]. In a similar study by Aldosari and Thomassin, a mean postoperative ABG of 20 dB after incus interposition was obtained. Aldosari and Thomassin also performed endoscopic bone cement (Hydroxyapatite) ossiculoplasty for necrosed incus long process (including post-stapedectomy and chronic suppurative otitis media) and reported an ABG of 5 dB [26].

Throughout the literatures, different studies were carried out to compare incus interposition with rebridging with bone cement in management of defects of incus long process. Some studies found that hearing gain was statistically greater in rebridging with bone cement than in incus interposition [5–8]. Other studies, on the other hand, found that both techniques were effective, and no one was superior to the other [9–11]. Despite such variations between those studies, they all had a common feature were all of them were performed entirely microscopically.

To the best of our knowledge, our study is the first one in the literatures to compare between rebridging with bone cement and incus interposition endoscopically. In our study, hearing gain was statistically greater in rebridging with bone cement than in incus interposition. Air bone gap (ABG) closure within 20 dB was achieved in 81.6% and 71.1% of bone cement and incus interposition groups respectively. In addition, no serious complications; in the short term; were encountered with the use of bone cement. All these finding confirmed that rebridging with bone cement was superior to incus interposition in management of incus long process defects. Such observation was comparable to the previously mentioned studies which utilized the microscope [5–8].

Endoscopic ossiculoplasty has many advantages over the conventional microscopic type. It allows wide-angled panoramic visualization of all middle ear structures. This advantage is of importance in proper placement of the graft material, prosthesis, or bone cement application resulting in better hearing outcome. In addition, it enables an easier passage through narrow and tortuous external auditory canal. Moreover, it avoids postauricular incision and hence provides better cosmetic outcome and shorter recovery time [27–30]. The disadvantages of endoscopic ossiculoplasty are similar to that of endoscopic ear surgery including single hand technique, relatively long learning curve and lack of stereoscopic view [31–33]. However, such obstacles were overcomed in the recent years. The obstacle of single hand technique seems to be more apparent during endoscopic incus interposition because incus interposition is difficult to be performed using single hand. Endoscopic rebridging with bone cement, on the other hand, can be easily performed using single hand while the other hand is holding the endoscope. Single handed technique and relatively long learning curve were overcomed by adequate endoscopic ear surgery training [31, 32].

The main limitation of this study was the short follow-up period. Further studies with relatively long-term follow-up are strongly recommended.

## Conclusion

Endoscopic transcanal rebridging with bone cement offers greater hearing gain than endoscopic transcanal incus interposition. The two techniques remain reliable and cost-effective techniques in management of patients with incus long process defects.
